# Combination of consensus and ensemble docking strategies for the discovery of human dihydroorotate dehydrogenase inhibitors

**DOI:** 10.1038/s41598-021-91069-7

**Published:** 2021-06-01

**Authors:** Garri Chilingaryan, Narek Abelyan, Arsen Sargsyan, Karen Nazaryan, Andre Serobian, Hovakim Zakaryan

**Affiliations:** 1 Institute of Molecular Biology of NAS RA, 0014 Yerevan, Armenia; 2 Russian-Armenian University, 0051 Yerevan, Armenia; 3 Denovo Sciences, 0033 Yerevan, Armenia; 4 Advanced Solutions Center, Foundation for Armenian Science and Technology, 0033 Yerevan, Armenia

**Keywords:** Virtual drug screening, Virtual screening

## Abstract

The inconsistencies in the performance of the virtual screening (VS) process, depending on the used software and structural conformation of the protein, is a challenging issue in the drug design and discovery field. Varying performance, especially in terms of early recognition of the potential hit compounds, negatively affects the whole process and leads to unnecessary waste of the time and resources. Appropriate application of the ensemble docking and consensus-scoring approaches can significantly increase reliability of the VS results. Dihydroorotate dehydrogenase (DHODH) is a key enzyme in the pyrimidine biosynthesis pathway. It is considered as a valuable therapeutic target in cancer, autoimmune and viral diseases. Based on the conducted benchmark study and analysis of the effect of different combinations of the applied methods and approaches, here we suggested a structure-based virtual screening (SBVS) workflow that can be used to increase the reliability of VS.

## Introduction

**Figure 1:**
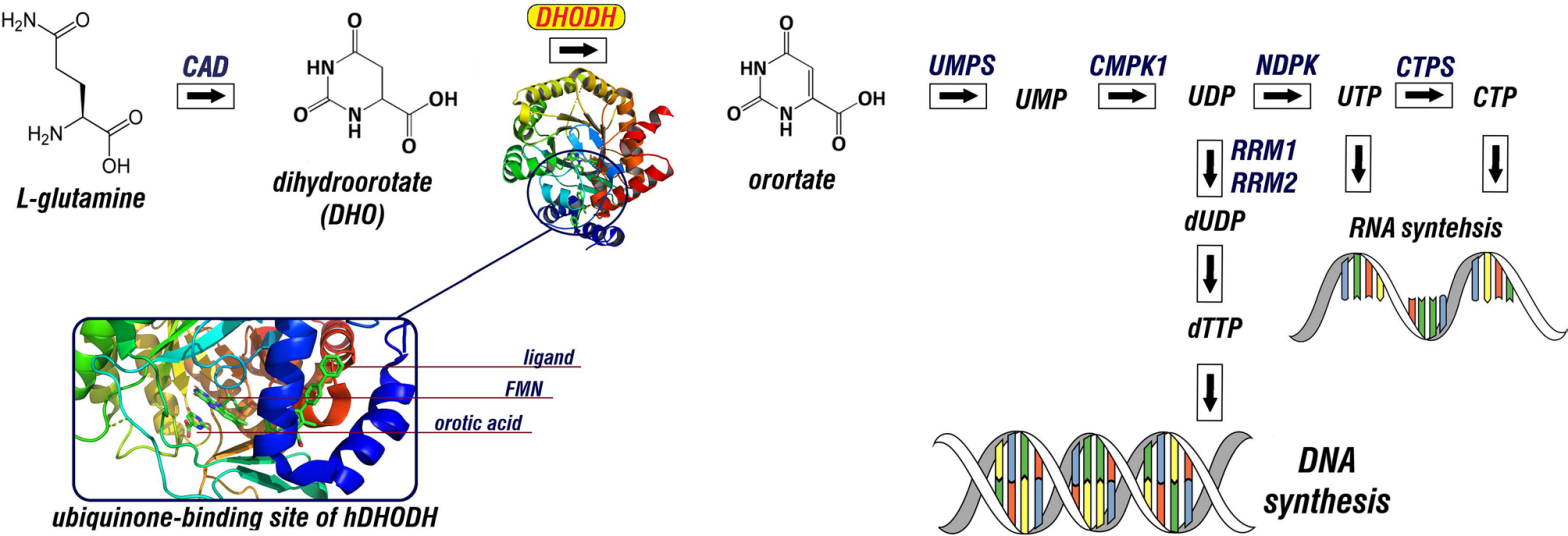
Schematic representation of de novo pyrimidine biosynthesis pathway and the 3D structure of hDHODH and the ubiquinone binding site. PyMOL v. 2.3.2 (https://pymol.org) was used to visualize the structure of *h*DHODH in complex with FMN and orotate (PDB ID: 1D3G). Schematic illustration was drawn using GIMP v 2.10.22 (https://www.gimp.org/).

The hDHODH is a rate-limiting enzyme of the de novo pyrimidine synthesis pathway. The enzyme catalyzes the oxidation of dihydroorotate to orotate, which is essential for the production of uridine monophosphate^1,2^ (Fig. 1). Inhibition of hDHODH results in pyrimidine depletion, thereby starving the cell of the essential nucleotides required in the cell cycle. In humans, DHODH has been widely recognized as a promising target for the treatment of cancer and autoimmune diseases^3,4^. Furthermore, inhibitors of hDHODH are of special interest as host-targeting broad-spectrum antiviral agents against a wide variety of DNA and RNA viruses^5–8^. In the context of the ongoing pandemic of severe acute respiratory syndrome coronavirus 2 (SARS-CoV-2), referred to as coronavirus disease 2019 (COVID-19), hDHODH inhibitors are considered as potential host targeting antiviral treatment of COVID-19^7–9^. Therefore, hDHODH is of exclusive interest as a potential therapeutic target for both treatment of the COVID-19 and potential prevention of the epidemics and pandemics occasionally caused by the emerging and re-emerging viruses. Given the medical significance, there are ongoing efforts towards the discovery and development of selective inhibitors for hDHODH.

Structure-based virtual screening is an established powerful computational approach that is extensively used in drug design and discovery due to its dramatic time-, cost- and labor-savings^10^. At the time of this publication, the Protein Data Bank^11^ (PDB) contained more than 60 crystal structures of hDHODH determined in the holo form, complexed with substrates, or inhibitors. The current structural data sufficiently allows researchers to conduct VS experiments in order to find new selective inhibitors for hDHODH^8,12,13^. However, based on the binding pose prediction success rates in the recent benchmark study, hDHODH has been characterized as a “very hard” and challenging molecular target for docking14. Furthermore, a number of open-source academic and commercial molecular docking software packages show quite varied performances depending on protein family and also on crystal structure of the protein^14–21^. In this context, the traditional approach in using a single software and structural conformation to screen a large database of molecules may lead to the questionable results.

Considering the therapeutic value of hDHODH, we have decided to develop a manual VS workflow, that integrates several known methods and approaches for the enhancement of VS efficiency, in particular ensemble and consensus docking strategies^22–30^. In order to validate this newly developed approach, we conducted a benchmark study. While we developed and tested SBVS workflow for hDHODH, we also tested and discussed its general application and efficiency.

## Results and discussion

### Selection of the representative set of the hDHODH ubiquinone binding site structural conformations

**Figure 2:**
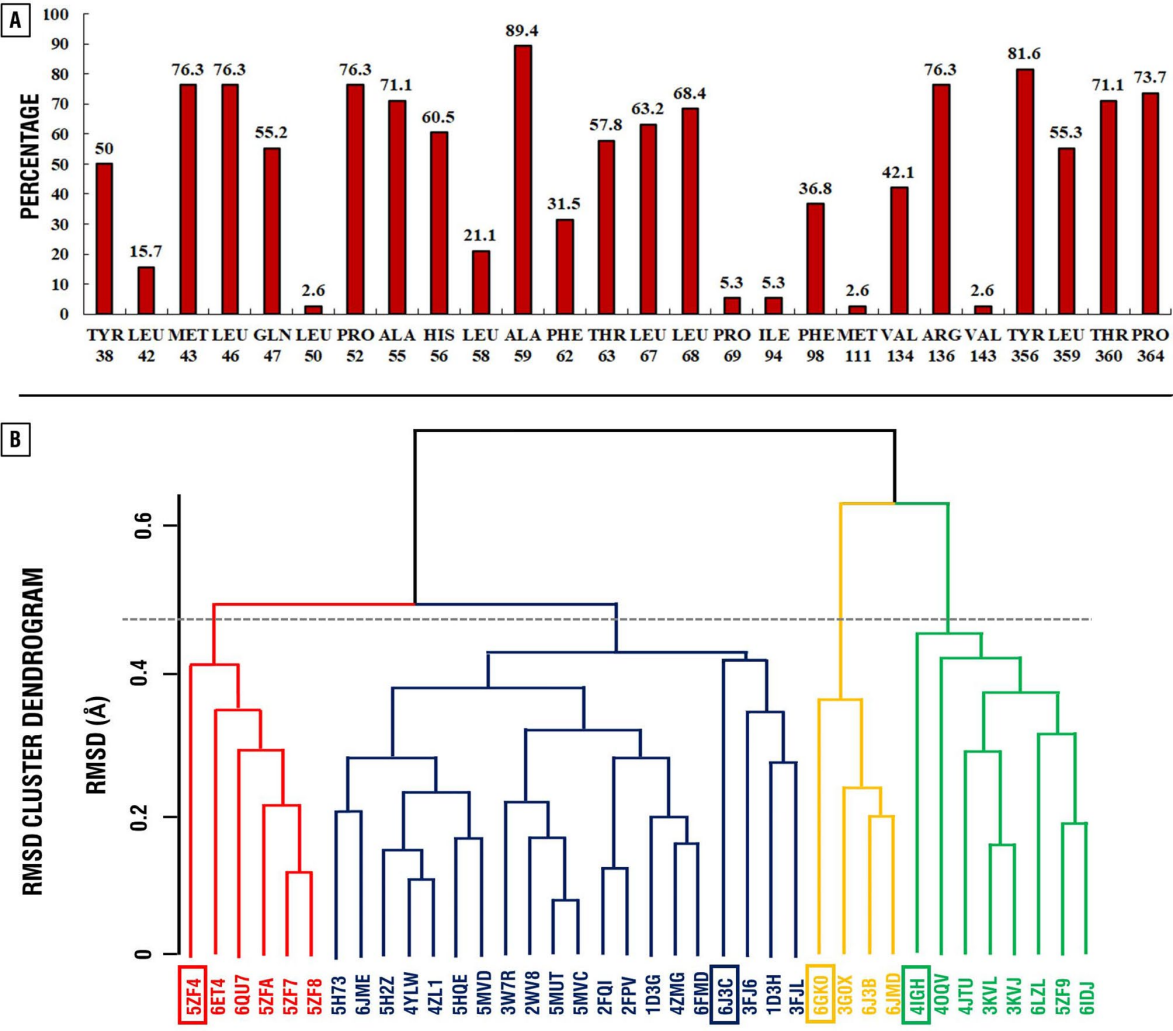
Distribution of interacting amino acid residues of the ubiquinone binding site of hDHODH among 38 crystal structures in percentage (**A**) and their clusterization based on the RMSD (**B**). Histogram was obtained using Microsoft Excel 2019. RMSD cluster dendrogram was obtained using Bio3D^31^ v2.3–0 (thegrantlab.org/bio3d_v2/).

Information on the interacting amino acid residues of the ubiquinone binding site of hDHODH (Fig. 2A) was gathered from the 38 selected crystal structures of hDHODH complexed with inhibitors (X-ray resolution < 2 Å). As a result of clusterization based on RMS deviation of the interacting amino acid residues of the ubiquinone binding site of hDHODH, four clusters were selected (cutoff 0.45 Å) (Fig. 2B). For each cluster, a representative structure was selected (PDB IDs: 4IGH, 6GK0, 6J3C, 5ZF4).

### Virtual screening

**Figure 3:**
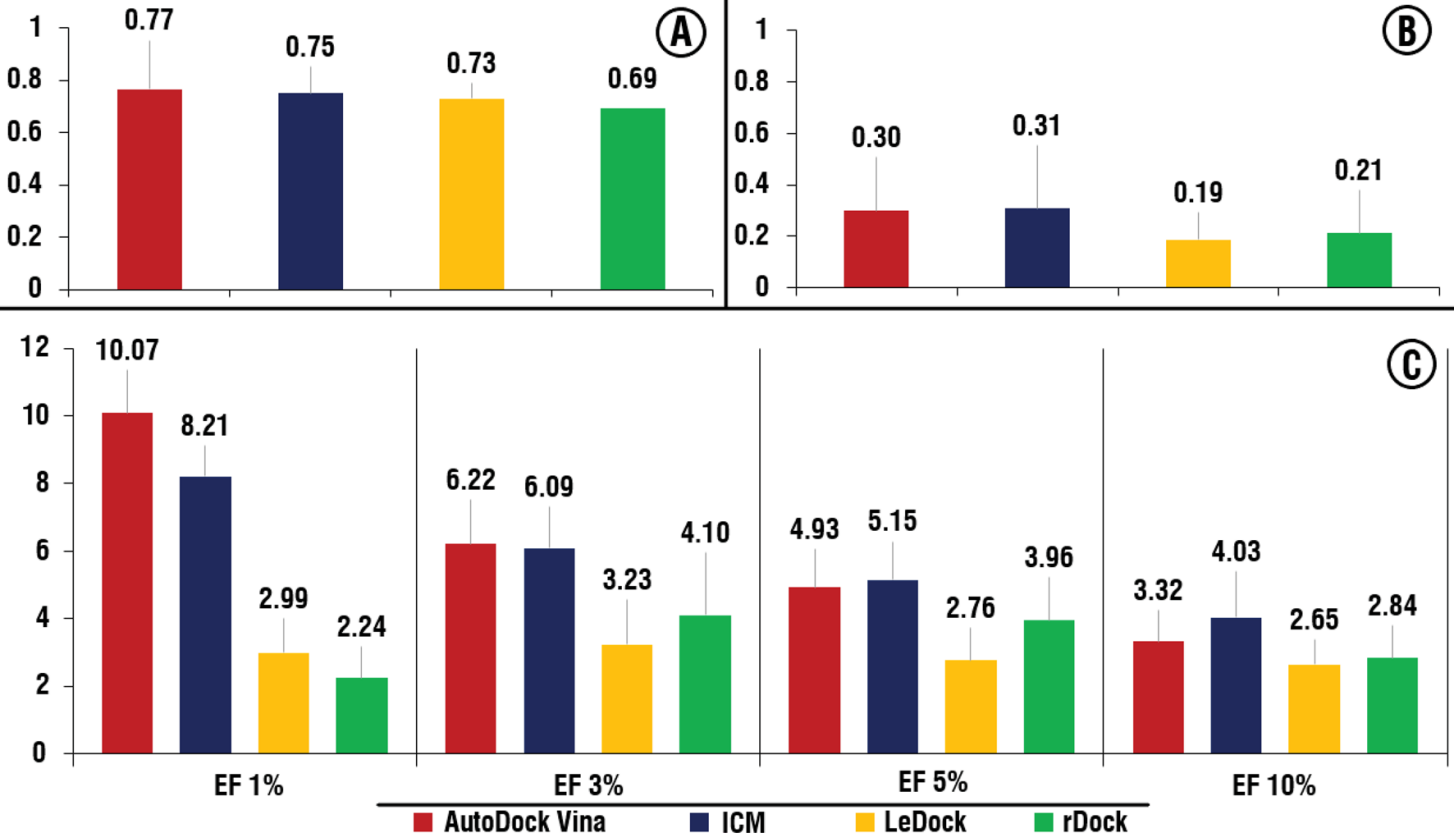
Average performances of the AutoDock Vina, ICM LeDock and rDock among four structures. (**A**) AUC, (**B**) BEDROC 20, (**C**) EF for 1, 3, 5 and 10%. Histograms was obtained using Microsoft Excel 2019.

We performed virtual screening of the benchmarking library for four software against four selected representative crystal structures of the hDHODH ubiquinone binding site (16 runs, Supplementary Table S2). Based on these separate 16 runs, the range of the values for AUC was from 0.62 to 0.84, for BEDROC 20 from 0.09 to 0.49 and for EFs (1%) from 1.49 to 14.93, EF (3%) from 1 to10.95, EF (5%) from 1.19 to 8.66, EF (10%) from 1.19 to 6.12 (Supplementary Table S3). The obtained data confirmed that performance of the SBVS procedure highly depends on the selected molecular docking software and representative structure of the protein. Based on the average value among four used structural conformations of hDHODH, AutoDock Vina and ICM demonstrated close and significantly better results than the remaining two software, LeDock and rDock (Fig. 3).

Taken together, obtained results showed that the performance of virtual screening may significantly vary depending on the structural conformation of hDHODH. In case of AutoDock Vina, ICM and LeDock screening against 4IGH and 6J3C crystal structures resulted in relatively better performance, compared to other structures, while for rDock—5ZF4 was relatively better structure (Supplementary Table S3).

### Ensemble docking and consensus-scoring

**Figure 4:**
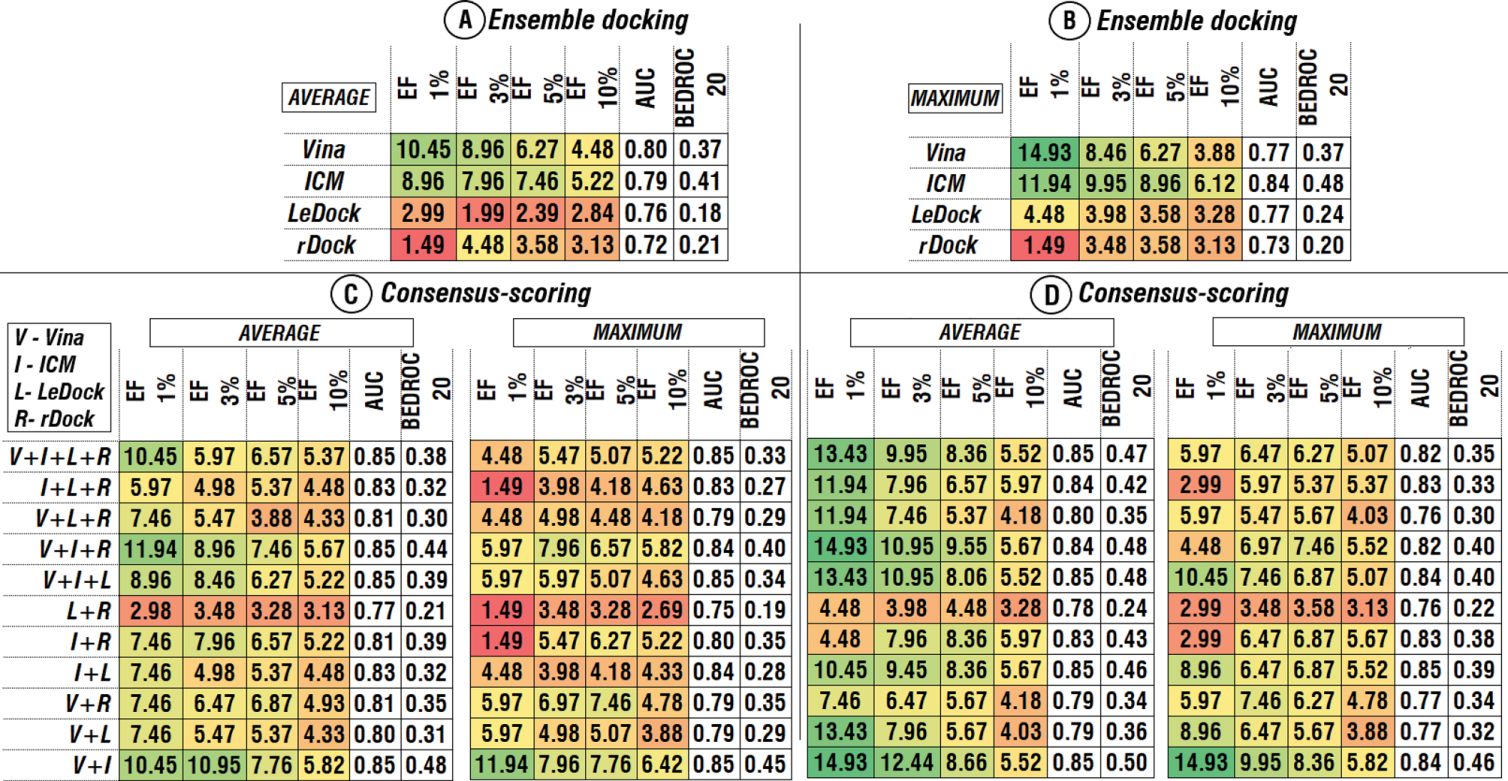
AUC, BEDROC 20 and EFs of different combinations of consensus-scoring and ensemble approaches. Combination of docking scores among four structures for separate software, based on the average (**A**) and the maximum (**B**) docking score values. Consensus-scoring in case of different software combinations (**C**,**D**). Colors correspond to the heatmap of the EFs values among combinations. Heatmap was obtained using Microsoft Excel 2019.

**Figure 5:**
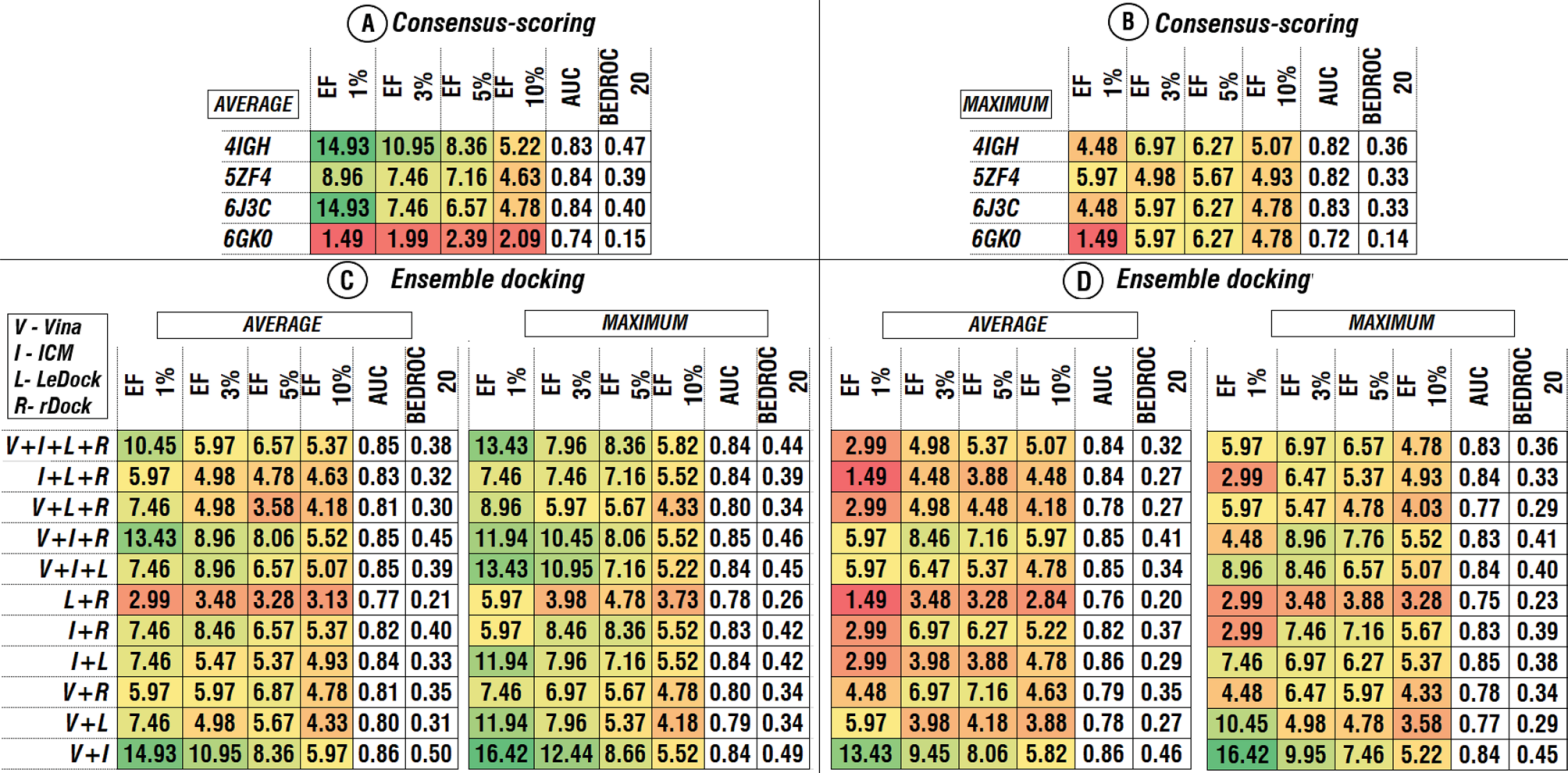
AUC, BEDROC 20 and EFs of different combinations of consensus-scoring and ensemble approaches. Combination of docking scores among four software for separate structures, based on the average (**A**) and the maximum (**B**) docking score values. Ensemble docking among four structures in case of different software combinations (**C**,**D**). Colors correspond to the heatmap of the EFs values among combinations. Heatmap was obtained using Microsoft Excel 2019.

Another important issue is multiple possible combinations of ensemble docking and consensus-scoring approaches and the difference in their effect on the improvement of the VS procedure. For the combination of ensemble docking and consensus-scoring approaches two scenarios were tested: (1) Docking scores were combined among four structures for separate software, then consensus-scoring was used among different software (Fig. 4) and conversely (2) A consensus-scoring approach is implemented among different software for separate structures, then normalized scores were combined among all structures (Fig. 5).

In the first case, a combination of ensemble docking approach based on the maximum docking scores (Fig. 4B) with the following consensus-scoring approach based on the average scores (Fig. 4D, “average”) demonstrated relatively better among all tested combinations of software. This combination showed the most stable positive effect among all tested different combinations of ensemble and consensus-scoring approaches (Figs. 4 and 5). In the second case, combinations based on the average score values among software (Fig. 5A) followed by combinations based on the maximum scores demonstrated better results than other combinations (Fig. 5C, “maximum”).

Among all possible combinations of software, the combination of the ICM and AutoDock Vina demonstrated the best results based on the all evaluated metrics (Figs. 4 and 5).

The effect of the different combinations of the ensemble and consensus-scoring approaches on the outcome of the virtual screening varied highly, depending on the way those approaches were combined, especially in the terms of enrichment factor. For the ICM and AutoDock Vina combinations, the range of the values of EF for 1, 3, 5 and 10 % are from 10.45 to 16.42, from 7.96 to 12.44, from 7.46 to 8.66 and from 5.22 to 6.42, respectively, while the differences of BEDROC 20 varies from 0.45 to 0.50 (Figs. 4 and 5, V +  I). For the combination of all four software EFs values varied from 2.99 to 13.43, from 4.98 to 9.95, from 5.07 to 8.36 and from 5.07 to 5.82 for EF 1, 3, 5 and 10 % respectively, while the BEDROC 20 values varies from 0.32 to 0.47 (Figs. 4 and 5, V +  I  + L + R). For those two combinations AUC values have not changed significantly (2–3 %). Taking into account the importance of early recognition of active compounds for real-life virtual screening application, two aforementioned best combinations in terms of enrichment factor were selected for further analysis.

**Figure 6:**
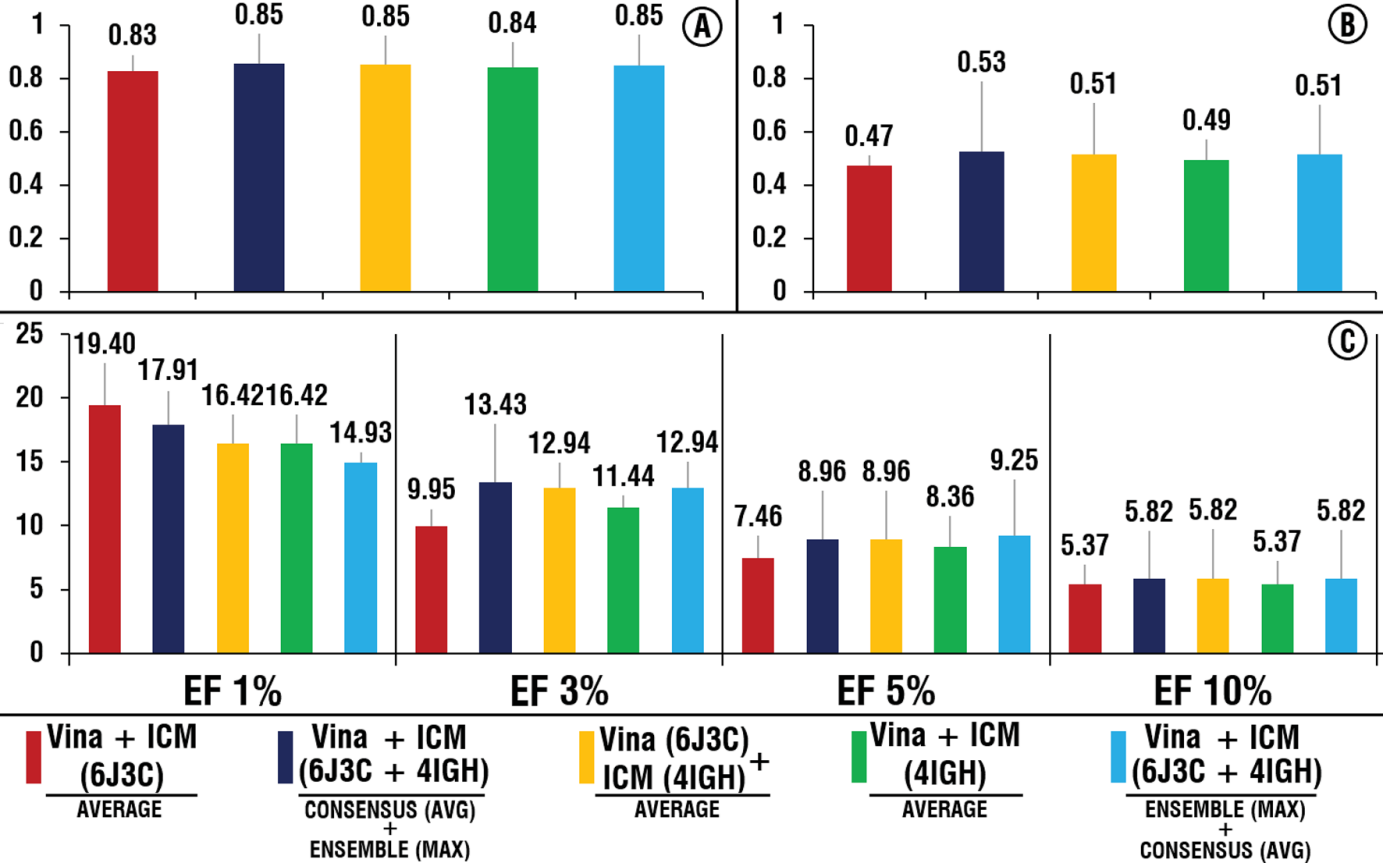
Performance of the selected combinations of consensus-scoring and ensemble approaches for AutoDock Vina and ICM in case of using two best structures (6J3C and 4IGH) separately and in combination. (**A**) AUC, (**B**) BEDROC 20, (**C**) EFs values. Histogram was obtained using Microsoft Excel 2019.

Since performance of VS software significantly varied for the different structural conformations of protein, we also studied the effect after elimination of “undesirable” structural conformations (conformations use of which resulted in relatively worse performance) of hDHODH for the AutoDock Vina and ICM combination of software (Fig. 6). Based on the initial separate VS runs, 6J3C and 4IGH were selected as the relatively best structural conformations (again based on the enrichment factor values) for the AutoDock Vina and ICM, respectively. We tested two scenarios, using the selected structures, in combination and separately (Fig. 6).

As seen in Fig. 6, compared to the scenarios when all four structures were applied (Figs. 4 and 5), elimination of undesirable structures has not significantly affected the performance and only in two cases values of EF (1 %) slightly increased: 1) when both 6J3C and 4IGH structures are used for both software and consensus-scoring approach based on the average score values followed by an ensemble approach where maximum score values are applied and 2) when only the 6J3C structure is considered for both software and consensus-scoring based on the average values are applied (Fig. 6). When only 6J3C structure is considered, values of EF (3 %) slightly increased for the first case and decreased for the second case (Fig. 6). Two points are needed to be noted here: (1) minor changes in the performance, after elimination of “undesirable” structures, are justified by the fact that previously applied ensemble and consensus-scoring approaches have already minimized the impact of those structures, and (2) these small differences, observed in cases of using different combinations of two best structures (6J3C and 4IGH), may be highly dependent on the set of active compounds of the dataset used and should not be taken into account for the actual VS procedure. This shows that elimination of “undesirable” structural conformations (Fig. 6) does not negatively affect the outcome of VS and linearly decreases computational time of VS procedure. It follows that, if it is possible to distinguish the reliability of the structures beforehand, it is reasonable to limit the selection of structural conformations to the most appropriate ones.

### Consensus binding poses

As the ensemble approach (multiple structures) was implemented, it is reasonable to evaluate success rates of software to predict crystal conformations of all 67 active compounds using selected representative crystal structures of hDHODH (Table 1). Based on our results (Table 1), all software performed reasonably well, with LeDock being most consistent among 4 crystal structures. Therefore, in addition to the methods used, we decided to apply an approach described by Houston and Walkinshaw^27^. These authors recommended to use several docking software to identify consensus binding poses for compounds, which increases reliability of predicted poses and consequently improve hit rates.

**Table1:** Success rates of binding pose predictions of crystal conformation of the 67 active compounds.

PDB IDs	Vina (%)	ICM (%)	rDock (%)	LeDock (%)
4IGH	82.08	85.07	79.1	82.08
5ZF4	76.12	82.08	79.1	82.08
6J3C	79.1	71.64	71.3	86.57
6GK0	71.64	79.1	77.61	77.61

We have tested the effect of the application of the consensus binding pose approach for the combination of all four software (Table 2) and combination of the AutoDock Vina and ICM (Table 3). In order to evaluate the effect of consensus binding approach in case of using different combinations of structural conformations, same scores (scores of the combination of ensemble docking based on the maximum scores of the 6J3C and 4IGH structures for Vina and ICM, separately, and following consensus-scoring among that software based on the average score values) for compounds were applied.

**Table 2:** Effect of the application of consensus binding pose filter in case of using combination of four used software (AutoDock Vina, ICM, LeDock, rDOCK).

Structures used for CBP	active/inactive	Percentage of active compounds in list (%)	Top 25	Top 50	Top 100
Initial dataset (no CBP)	67/1933	3.35	14	24	31
4 structures	27/350	7.16	10	16	19
4IGH + 6J3C	30/457	6.16	12	19	21
6J3C	36/609	5.58	14	17	25
4IGH	40/758	5.01	14	19	26
5ZF4	37/745	4.73	14	17	25
6GK0	40/851	4.49	14	18	27

**Table 3:** Effect of the application of consensus binding pose filter in case of using combination of the AutoDock Vina and ICM.

Structures used for CBP	active/inactive	Percentage of active compounds in list (%)	Top 25	Top 50	Top 100
Initial dataset (no CBP)	67/1933	3.35	14	24	31
4 structures	42/738	5.39	16	22	29
4IGH + 6J3C	49/989	4.72	17	24	33
6J3C	54/1236	4.19	13	22	28
4IGH	59/1413	4.01	15	24	30

As seen in Tables 2 and 3, inclusion of the additional software and structures affected the ratio of active to inactive compounds in a positive way for both cases. In case of using all available software for filtering with the CBP approach (Table 2), a number of active compounds in the top of the ranked list, along with inclusion of additional structures, noticeably decreases compared to the initial dataset. In contrast, by using the best combination of software the difference between using all four, two and one structure is less significant compared to the initial dataset and in some cases number of compounds actually increases (Table 3).

Therefore, if there is no pre-validated data on software and structures performance and one is not able to distinguish their selection to the most appropriate ones, then it is reasonable not to use multiple structures of protein for the filtering with the CBP approach. Despite the fact that use of multiple structures for the filtering with the CBP approach may increase the percentage of potential hit compounds, unregulated elimination of some active compounds, can negatively affect virtual screening procedure in terms of early recognition. However, when one is able to conduct a manual benchmark study for the investigated protein beforehand, or has pre-validated data on the performance and “suitability” of selected software and structural conformations of the protein, limitation of both software and structures to the most appropriate ones is advantageous. Alongside with the increase in the percentage of potential active compounds in the library, application of such a filter can positively affect VS procedure in terms of early recognition, by additional placement of the active compounds in the top of the ranked list.

**Figure 7:**
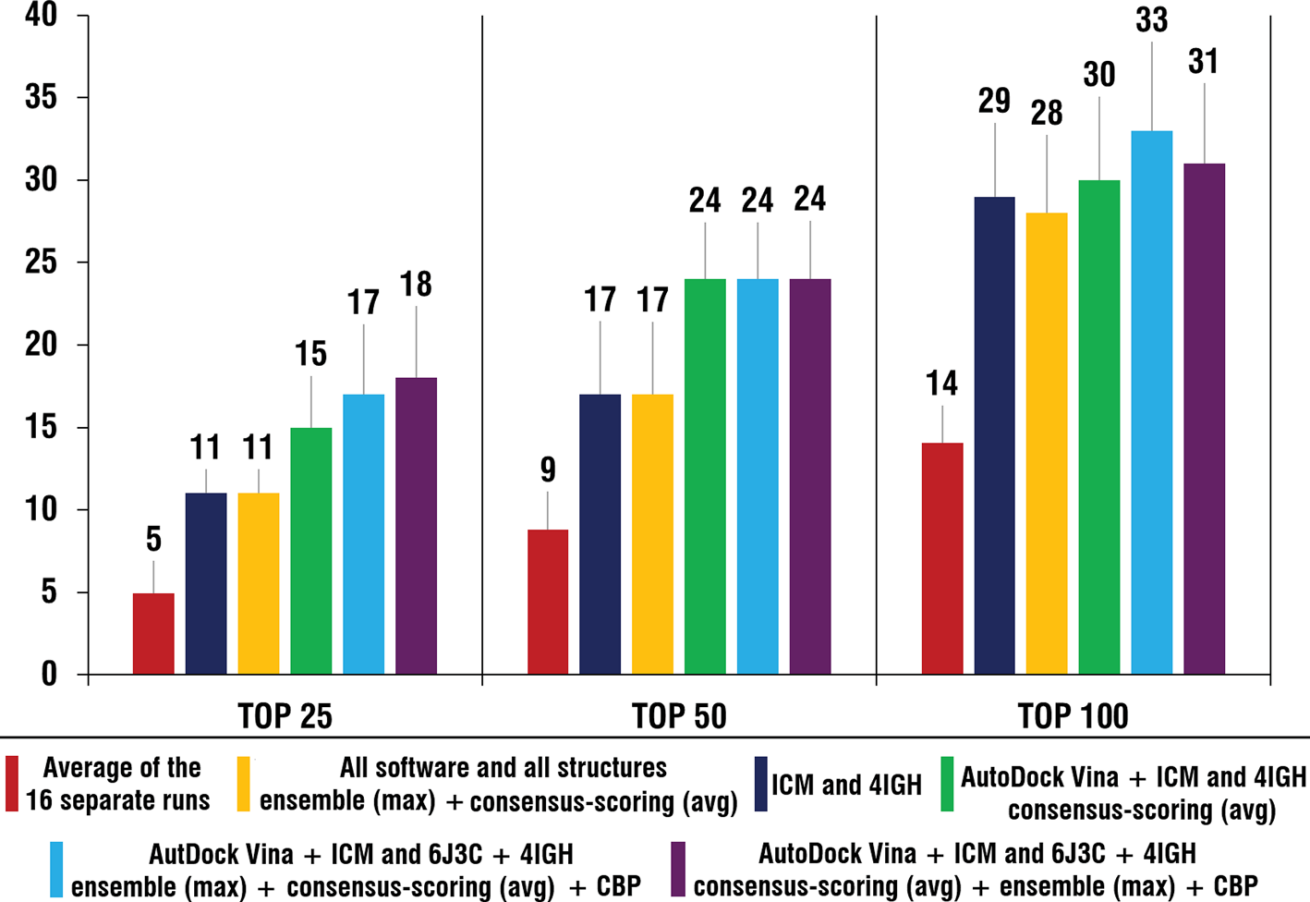
Comparison of the number of active compounds in the top 25 (1.25%), 50 (2.5%) and 100 (5%) of the ranked lists after application of the best identified combinations of the ensemble and consensus approaches to the average number of compounds (based on the 16 separate runs) and best “one software—one structure” case (ICM and 4IGH). Histogram was obtained using Microsoft Excel 2019.

The main effect of the application of the ensemble and consensus docking approaches is in the increase of the reliability of VS results. As seen in Fig. 7, even in the case when pre-validated data is not taken into account and all available software and structural conformations of protein are used, the outcome (number of active compounds in top of the list) is similar to that of the best “one software—one structure” performance. However, if pre-validated data is available application of those approaches can significantly improve the performance of VS procedure, especially in terms of early recognition of potential hit compounds (Fig. 7).

### Performance and applicability of the proposed SBVS workflow

Although an inclusion of the additional software and the use of multiple structural conformations of the investigated protein increases the computational time, it does not dramatically affect the overall time of the virtual screening procedure. We used a local workstation with Threadripper 3970 × CPU (32 cores, 64 threads) and calculations of the library of 2000 compounds using proposed workflow took ~ 1.5 h to complete, calculations of the library of 500,000 compounds (unpublished data) took ~ 2 weeks. The proposed SBVS workflow is scalable and applicable for any library if enough time or computational resources are available, including ultra-large virtual screening procedures, when usually high-performance computing systems with ~ 1000 s of CPU cores are used.

## Conclusion

**Figure 8:**
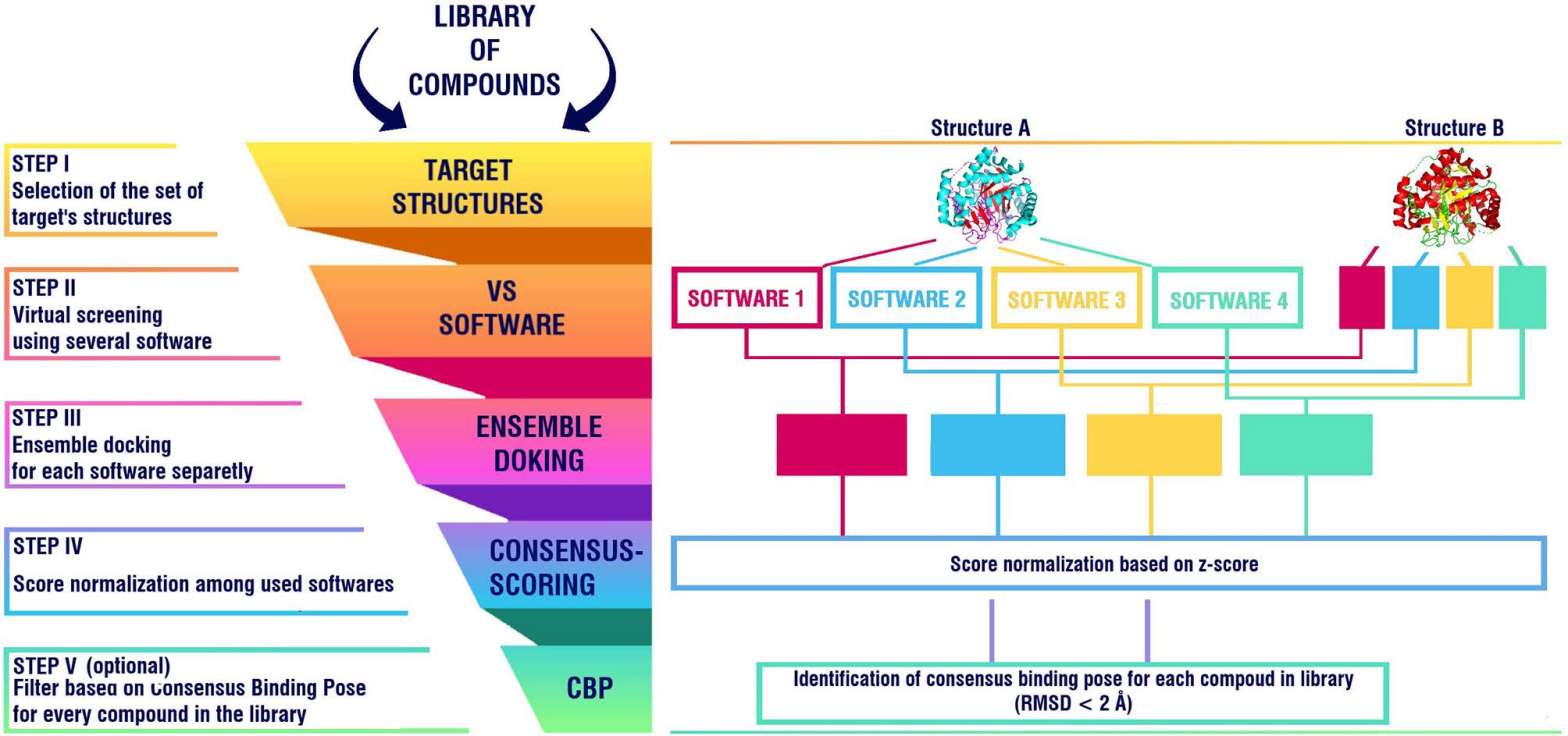
Schematic representation of the developed SBVS workflow. Schematic illustration was drawn using GIMP v 2.10.22 (https://www.gimp.org/). 3D structures of “Strucutre A” (*h*DHODH, PDB ID: 6J3C) and “Structure B” (*h*DHODH, PDB ID: 4IGH) were obtained using PyMOL v. 2.3.2 (https://pymol.org).

Taking into the account inconsistencies in the performance of the VS process, depending on the software and structural conformations of protein, appropriate application of the ensemble and consensus docking approaches significantly increases reliability of the VS results. However, their inappropriate integration can lead to negative outcomes in terms of performance. Based on the conducted benchmark and analysis of the different combinations of the applied methods and approaches, here we suggested a SBVS workflow that can be used to increase the reliability of the VS procedure (Fig. 8). In cases, like ours, when one is able to use pre-validated data, and limit the selection of the software and structures to the most appropriate ones, use of such workflow can increase the performance of the VS procedure in terms of early recognition of the potential hit compounds, which is of paramount importance in real-world screening applications, where researchers test top-ranked molecules in biological assays due to their high costs. Therefore, pre-validation of docking software and protein structures, before the actual VS procedure, is highly recommended.

## Methods

### Structure selection and preparation

38 crystal structures of hDHODH in complex with inhibitors (resolution < 2 Å) have been selected from the Protein Data Bank (PDB) (Supplementary Table S1). Ligands, water, metal ions and other co-crystallized molecules were removed, except co-factor of hDHODH—flavin mononucleotide (FMN). Then protein structures were superimposed using ICM-browser^32^. For the selection of a representative set of structures, clusterization method based on root-mean-square deviation (RMSD) of the interacting amino acid residues (Cα atoms) of ubiquinone binding site of hDHODH was performed using bio3D R package^31^. Pairwise approach was used for the RMSD calculations among 38 selected structures. Important amino acid residues of the ubiquinone binding site were selected based on the distribution of the interacting amino acids with the co-crystallized ligands among 38 used crystal structures (based on the annotations of crystal complexes) (Fig. 2A). Polar hydrogen atoms were added to the structures with the protonation state at pH = 7, using Open Babel 2.4.0 software^33^.

### Benchmarking dataset and evaluation metrics

Directory of Useful Decoys: Enhanced^34^ (DUD-E) dataset of decoys for the hDHODH was downloaded, filtered for duplicates and 1933 unique decoys were selected. 67 crystal structures of hDHODH in complexes with inhibitors, including 38 complexes used for the selection of target’s representative structures, were downloaded from the PDB^11^ (Table S1). Co-crystallized ligands were extracted from corresponding complexes and used as active molecules for the benchmark study. Therefore, the final benchmarking library consisted of 67 active compounds and 1933 decoys (inactive compounds).

Several commonly used metrics, such as area under the ROC curve (AUC), Boltzmann-enhanced discrimination of ROC (BEDROC, with α = 20), and enrichment factors (EF) for several percentage were used to evaluate and compare accuracy and performance of the conducted virtual screening procedures and calculated using rocker^35^. Enrichment factor was calculated for 1, 3, 5 and 10%. For the evaluation and comparison of the effects of applied approaches and selection of relatively best structural conformations for individual software, the preference is given to the enrichment factor as a selection criterion. Other general metrics, such as ROC AUC, have difficulties to characterize the initial part of ranked lists (two VS methods with similar values of AUC can have significantly different numbers of active compounds in the top of the ranked list)^36–38^. Since we applied a filter that changes the ratio of active to inactive compounds of the initial dataset and makes direct comparison of aforementioned metrics inaccurate, as an additional comparative metric we simply compared the numbers of active compounds found in the top 25, 50 and 100 of the ranked lists.

### Docking software and parameters

Four molecular docking software packages were used, including three open-source/freely-distributed (AutoDock Vina^39^, LeDock^40^ and rDOCK^41^) and one commercial (ICM^32^) packages. The selection of VS software was based on the several factors, such as high mean general performance, differences in pose prediction and scoring methods, availability of commercial software in our laboratory, etc. Other VS software and their combinations can be evaluated following described methodology. Main differences in molecular docking software are search algorithms and scoring functions. LeDock uses evolutionary algorithm adopted in combination with simulated annealing search, which is used to generate the first generation of docking poses and physics and knowledge-based hybrid scoring function^40^. RDock uses combination of stochastic and deterministic search techniques to generate low energy ligand poses and rDock master scoring function, which is a weighted sum of intermolecular, ligand intramolecular, site intramolecular, and external restraint terms^41^. The ICM docking algorithm is a global energy optimization procedure based on Monte Carlo minimization^32^. ICM has 2 scores: "ICM Score" and Potential of Mean Force (PMF) score. Only the ICM score, which is an empirical scoring function based on calculation of physiochemical properties of the receptor-ligand complex, was used in our study. AutoDock Vina uses "Iterated Local Search global optimizer" similar to that of ICM and Broyden-Fletcher-Goldfarb-Shanno (BFGS) quasi-Newton method for the local optimization for generation of docking poses and hybrid scoring function (empirical + knowledge-based)^39^.

The 67 crystal complexes of hDHODH with co-crystallized ligands were superimposed using icm-browser and docking box that covers all 67 ligands, based on their crystal structure conformations, and the amino acid residues of the ubiquinone binding site was used for AutoDock Vina and LeDock runs. In the case of the rDock, cavity mapping method based on the reference ligand was used. In the case of the ICM software, the docking grid was based on the interacting amino acid residues of the ubiquinone binding pocket of hDHODH (Fig. 2A). For all software packages, corresponding standard protocols were used with exception of rDOCK where the number of docking runs was set to 150.

### Ensemble docking and consensus-scoring

In ensemble docking scores of each compound, obtained from docking runs using the same software but different structural conformers of a target protein, are combined using different methods^24^. In consensus-scoring scores of each compound, obtained from individual software for the same representative structure of protein, are combined using different methods^28^. Since various docking software are used (i.e., different scoring functions), it is required to perform preceding score normalization. For the normalization of docking scores, from different software simple z-score function: z = (x –μ) / σ, where x is a compound score, μ and σ—mean and standard deviation of scores of all compounds in the library, respectively, was used. For, ensemble docking and consensus-scoring approaches, docking scores were combined using two common data fusion methods: based on average and maximum (lowest binding energy) score values^42,43^. We combined ensemble and consensus-scoring approaches by two alternative methods. 1) Docking scores of each compound obtained by one software for four structures were combined. Then, scores obtained by different software were normalized and also combined. 2) Docking scores of compounds for each structural conformation of hDHODH obtained by different software were normalized and combined. Then, new normalized scores of each compound were combined among four structures.

### Consensus binding pose approach (CBP)

An approach, based on consensus binding pose prediction and its use as a filter, described by Houston and Walkinshaw^27^, was implemented. The difference in our case is use of multiple structural conformations of the target protein. After molecular docking runs, the top ranked docked conformation of each compound was selected. Those conformations were used for consensus binding poses evaluation. Two poses were regarded as similar (consensus) if the RMSD value was below 2 Å, which is a widely accepted value in docking predictions^27,44,45^. All possible combinations of used docking programs were tested. Compounds, for which consensus binding poses for one structural conformation of protein were predicted by different software, passed into the new lists. Then those lists (one for each structure) were compared with each other, and if a compound was presented in all of the lists, then it was selected into the final filtered list.

## Supplementary Information

Supplementary Table S1

Supplementary Table S2

Supplementary Table S3
